# Physiological and molecular mechanisms of drought adaptation in foxtail millet: insights and future perspectives

**DOI:** 10.3389/fpls.2025.1701035

**Published:** 2025-12-12

**Authors:** Hui Gao, Jiaxin Liu, Shen Li, Luming Zou, Yulu Wang, Lin Li, Ting Zhang, Ling Zhao, Genping Wang, Haoshan Zhang

**Affiliations:** 1 Institute of Millet Crops, Hebei Academy of Agriculture and Forestry Sciences/Key Laboratory of Genetic Improvement and Utilization for Featured Coarse Cereals (Co-construction by Ministry and Province), Ministry of Agriculture and Rural Affairs/National Foxtail Millet Improvement Center/Key Laboratory of Minor Cereal Crops of Hebei Province, Hebei Academy of Agriculture and Forestry Sciences, Shijiazhuang, China; 2 Hebei Key Laboratory of Crop Stress Biology, Department of Life Science and Technology, Institute of Wild Plant Resources Application, Hebei Normal University of Science and Technology, Qinhuangdao, China

**Keywords:** foxtail millet, drought adaptation, ABA signaling, epigenetics, germplasm diversity, signaling networks, genetic diversity

## Abstract

Drought stress poses a major challenge to global agriculture under accelerating climate change. Foxtail millet (*Setaria italica*), a C4 crop native to China, has emerged as both a coarse grain crop in arid regions and a model for studying drought adaptation. This mini review synthesizes recent advances in understanding the multi‐level drought response network of foxtail millet, encompassing root system remodeling, stomatal regulation, osmotic adjustment, and photosynthetic and metabolic reprogramming. These physiological processes are coordinated by interconnected signaling modules involving Ca²^+^, reactive oxygen species (ROS), and abscisic acid (ABA), and are transcriptionally fine‐tuned by transcription factors (TFs), non‐coding RNAs, and epigenetic modifications. We also emphasize the genetic and germplasm diversity underlying drought tolerance, highlighting foxtail millet’s potential as a comparative C4 model for functional genomics and climate‐resilient breeding. Despite substantial progress, critical gaps remain in understanding hormone crosstalk, root–shoot signaling, and the integration of metabolic and transcriptional responses. Future research integrating pan‐genomics, multi‐omics, and precision genome editing, combined with translational breeding aimed at enhancing yield stability under climate variability, will deepen mechanistic understanding and accelerate the improvement of drought‐resilient cereal crops.

## 1 Introduction

Drought is a pervasive abiotic stress that severely limits agricultural productivity worldwide (FAO, 2023). Over the past two decades, climate change has intensified both the frequency and severity of drought events, leading to substantial yield losses in cereals such as wheat, maize, and rice (Nations, 2022). Global estimates suggest that more than half of the world’s population will be exposed to water scarcity (Mekonnen and Hoekstra, 2016; Veldkamp et al., 2016), making the development of drought-resilient crops a critical priority for food security. In China, arid/semi-arid regions cover over half the land area, with frequent drought disasters causing heavy economic losses (Wang et al., 2004; Zhai and Liu, 2012). Within this context, foxtail millet has gained prominence given its drought resilience (Sun et al., 2011; Hu et al., 2018; Pardo and VanBuren, 2021; Xiao et al., 2021; Zhao et al., 2025a).

Long-term breeding and cultivation of foxtail millet in arid regions has selected for drought-resilient traits, which enable foxtail millet to exhibit superior water-use efficiency and carbon fixation capacity under limited moisture compared to other C4 plants like maize and sorghum. Foxtail millet’s leaf water-use efficiency is more than twice that of maize and sorghum (Sun et al., 2011). Compared to maize, it uses roughly 70% of the water required to produce equivalent biomass (Niu and Guo, 2022). In addition, rich germplasm resources, high-quality reference genomes, multi-omics datasets, and mature transgenic technology make foxtail millet a new ideal platform for functional genomics and systems biology researchers (Jia et al., 2013; Upadhyaya et al., 2015; Doust and Diao, 2017; Hu et al., 2018; Yang et al., 2020; Li et al., 2022; He et al., 2023a).

In general, compared with economically important crops such as maize, wheat, and rice, foxtail millet has stronger drought adaptability and complete omics information, making it an ideal C4 model crop for drought research (Ceasar et al., 2025). In this mini review, we synthesize recent advances in foxtail millet drought adaptation, which centers on water acquisition and root system remodeling; stomatal regulation and water-use efficiency; osmotic adjustment and cellular protection; photosynthetic acclimation and energy metabolism; and signal perception and transcriptional reprogramming. We also discuss the uniqueness of foxtail millet as a comparative model for C4 drought biology and propose a conceptual framework for future integrative research.

## 2 Drought adaptation strategies in foxtail millet

**Figure 1:**
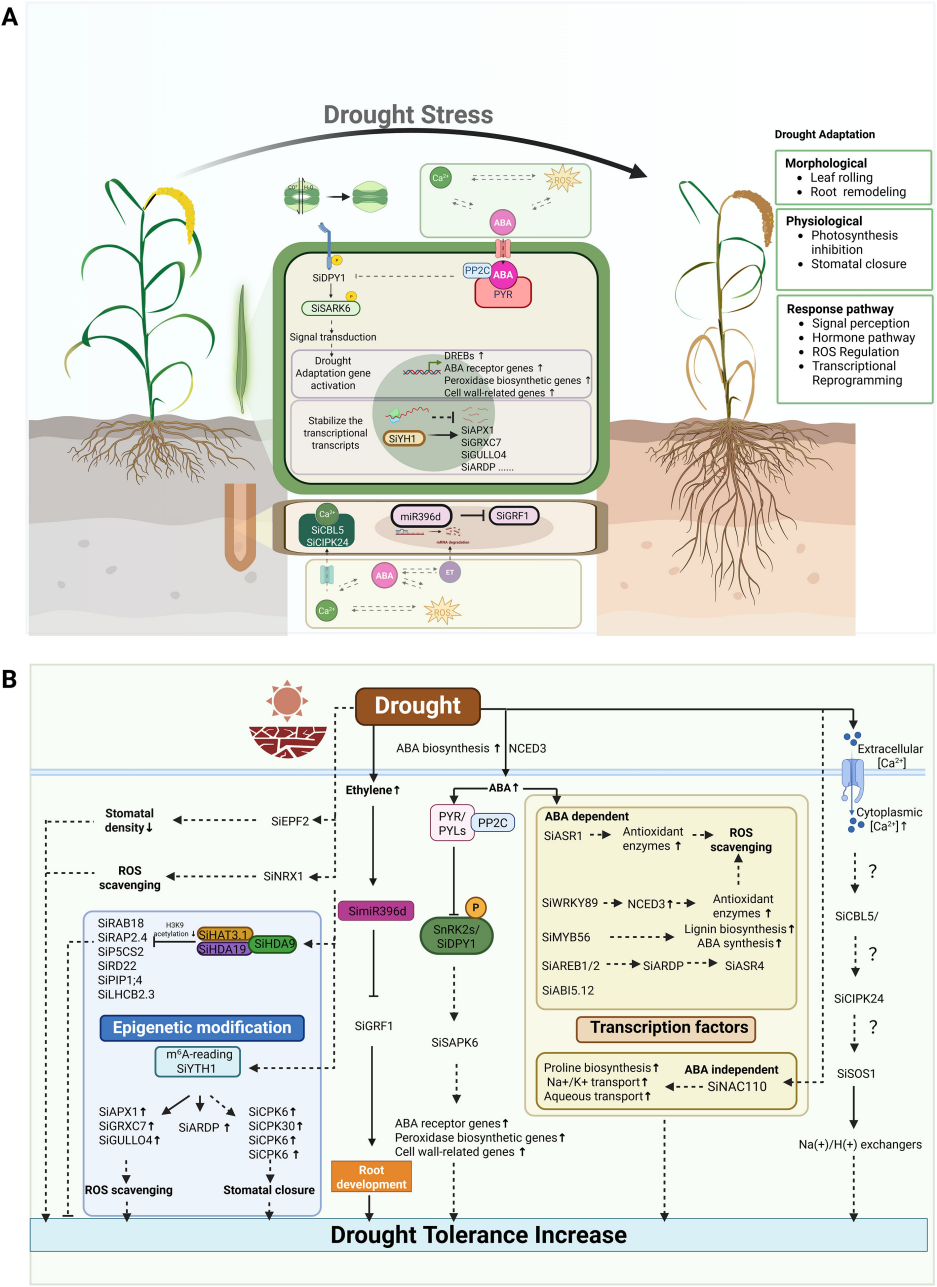
Integrated physiological and molecular mechanisms underlying drought adaptation in foxtail millet. **(A)** Physiological and signaling overview of drought stress adaptation in foxtail millet. Drought triggers a coordinated response involving root system remodeling, stomatal regulation, and photosynthetic adjustment. Calcium (Ca^2+^), reactive oxygen species (ROS), and abscisic acid (ABA) signaling converge to activate stress-responsive pathways in both roots and leaves. Key modules such as SiCBL5-SiCIPK24, SiDPY1-SiSAPK6, and SiYTH1-mediated m6A regulation coordinate drought perception, signal transduction, and transcriptional reprogramming. These interactions enhance root water uptake, promote stomatal closure, and maintain cellular homeostasis, collectively supporting morphological (leaf rolling, root remodeling) and physiological (photosynthesis inhibition, water-use efficiency) drought adaptation. **(B)** Network-level molecular regulation of drought tolerance in foxtail millet. At the mechanistic level, drought induces ABA biosynthesis (SiNCED3) and activates ABA–SnRK2–bZIP/NAC/WRKY modules, leading to the expression of genes for osmotic balance, ROS scavenging, and lignin biosynthesis. Cross-talk among ABA, ethylene, and Ca^2+^ signaling integrates with epigenetic and post-transcriptional regulation, including the miR396d–SiGRF1 module and SiYTH1-mediated m6A modification. Histone acetylation regulators (SiHDA9, SiHAT3.1, SiHDA19) further fine-tune gene activation. Together, these pathways establish a multi-layered regulatory network that enhances drought tolerance through integrated hormonal signaling, transcriptional control, and epigenetic modulation.

Foxtail millet exhibits a suite of finely coordinated drought-adaptation strategies that integrate morphological, anatomical, molecular, and biochemical adjustments. Foxtail millet is a short-statured crop with a low transpiration rate. It has a short growth cycle and matures quickly, effectively avoiding drought caused by climate change. As a small-grain crop, it tends to produce more seeds within a limited growing period (Sun et al., 2019; Ceasar et al., 2025). However, beyond simple avoidance, foxtail millet expresses dynamic physiological plasticity, which enabling rapid transitions between growth and conservation modes in response to fluctuating soil moisture. Although many of these strategies parallel those observed in other cereals, their expression in foxtail millet is typically faster, more reversible, and more tightly linked to molecular regulatory pathways, allowing efficient water uptake, minimized loss, and sustained metabolism during prolonged drought (**Figure 1A**).

### 2.1 Water acquisition and root system remodeling

Root system remodeling serves as the primary adaptive interface between foxtail millet and droughted soil environments. Foxtail millet possesses a dense, fibrous root system capable of extracting water from deep soil layers, forming the structural foundation for its drought tolerance (Ceasar et al., 2025). Comparative analyses across ecological regions demonstrate adaptive divergence in root traits. For example, genotypes from arid northwestern China develop significantly longer roots than those from humid northeastern regions, consistent with selective pressure from rainfall gradients (Yang et al., 2022). Under nutrient limitation, root architectural plasticity extends beyond water foraging, as foxtail millet enhances lateral root growth under phosphorus stress and thickens primary roots under nitrogen limitation responses that simultaneously improve hydraulic conductivity and drought resilience (Nadeem et al., 2020).

At the molecular level, drought perception in roots rapidly triggers cytosolic Ca²^+^ influx, which is decoded by Ca²^+^ sensors such as calcineurin B‐like proteins (CBLs) (Wilkins et al., 2016; Tang et al., 2020; de Oliveira et al., 2024; Qin et al., 2024). Among them, SiCBL5, a functional ortholog of AtCBL3, is highly expressed in roots and interacts with the kinase SiCIPK24 to regulate Na^+^ homeostasis and osmotic balance, thereby improving stress tolerance. Although overexpression of *SiCBL5* enhances drought resistance, the downstream network controlling root morphogenesis remains unresolved, highlighting a key mechanistic gap for future research (Yan et al., 2021). Proteomic analyses further indicate that root tissues exhibit broader activation of hormone‐responsive proteins than leaves, including SiMAPK (involved in ABA signaling) and SiOPR1 (linked to jasmonic acid biosynthesis), underscoring root‐specific drought signaling complexity (Gao et al., 2023).

In foxtail millet, the ethylene-dependent, drought-induced, and antagonistic SimiR396d-SiGRF1 module is a key molecular mechanism regulating drought tolerance and root remodeling. Seedlings of foxtail millet overexpressing *SimiR396d* exhibit elongated root length and enhanced drought tolerance; in contrast, seedlings with knockdown of *SimiR396d* or overexpressing *SiGRF1* in foxtail millet show drought-sensitive (Zhang et al., 2023). However, the drought phenotype of this module in the reproductive stage of foxtail millet has not been clarified, and its role in drought tolerance and stable yield of foxtail millet remains to be further evaluated (Zhang et al., 2023). Drought also induces genes involved in cell wall modification, cytokinin transport, and meristem differentiation, reflecting active remodeling of root growth zones. QTL mapping in interspecific crosses between *Setaria italica* and *Setaria. viridis* identified loci associated with root proliferation and cell cycle regulation under water deficit, confirming the developmental basis of root plasticity (Qie et al., 2014; Tang et al., 2017). These molecular adjustments collectively produce phenotypes characterized by accelerated root elongation, deeper rooting profiles, and enhanced rhizosheath formation (Ajithkumar and Panneerselvam, 2013; Liu et al., 2018; Gao et al., 2023). Transcriptomic data further reveal that primary energy metabolism pathways (glycolysis, TCA cycle, and ATP synthesis) are rapidly activated in roots during early drought stress, supporting the hypothesis that foxtail millet sustains active metabolic flux to fuel root growth and osmotic adjustment (Zhang et al., 2007). The root architecture thus transitions from shallow to steep, optimizing soil water extraction under drought stress (Xiong et al., 2025). Root water transport efficiency is enhanced via the upregulation of aquaporin genes (*SiPIPs*), which increase hydraulic conductivity and sustain shoot hydration (Zhang et al., 2007). Such physiological and molecular integration exemplifies the coordinated drought‐response hierarchy in foxtail millet, where signaling pathways (Ca²^+^, ethylene, and ABA) converge to remodel root architecture in a genotype‐and environment‐specific manner.

### 2.2 Stomatal regulation and water-use efficiency

Stomata are surrounded by a pair of guard cells and serve as the primary channels for plants to efficiently absorb carbon dioxide for photosynthesis, while also regulating water transpiration (Schroeder et al., 2001; Hsu et al., 2020; Liu et al., 2022a).

Stomatal movement is driven by ABA, Ca²^+^, and ROS signaling integration. Drought stress elevates ABA synthesis through SiNCED1 (Huang et al., 2023), leading to the activation of ABA receptors (SiPYLs) (Zhang et al., 2021; Ma et al., 2025), SnRK2 kinases (SiSAPK6) (Zhao et al., 2023), and TFs such as SiAREB/ABF and SiARDP, which coordinate stomatal closure and osmotic balance (Li et al., 2014, 2017). The m^6^A reader SiYTH1 stabilizes a set of drought-related transcripts, including SiARDP (an ABA-responsive DREB-binding protein) and ROS-scavenging genes such as SiAPX1, SiGRXC7, and SiGULLO4. This stabilization ensures signal continuity and fine-tuned stomatal kinetics, thereby enhancing Ca^2+^/ABA/ROS signaling for stomatal closure under drought stress. Under drought, *SiYTH1*-overexpressing seedlings exhibit >80% stomatal closure, which is significantly higher than the wild-type (~40%) and *siyth1* mutants (<25%) (Luo et al., 2023). Drought-tolerant foxtail millet cultivars have mainly evolved two distinct adaptive patterns of stomatal development. The first pattern is characterized by reduced stomatal density with no significant change in individual stomatal size, such as in the drought-tolerant variety (DTV) “MT3” (Hao et al., 2024). Previous studies have suggested that *EPFs*, which regulate stomatal density, are functionally conserved within Poaceae (Jangra et al., 2021). In foxtail millet, *siepf2* exhibit increased stomatal density, and greater drought sensitivity (Hao et al., 2024, 2025). The second pattern is high stomatal frequency combined with small stomatal area, enabling more flexible regulation of stomatal opening and closing to balance water and photosynthetic efficiency. For instance, the DTV “ISe-15” can induce a higher increase in ABA to promote stoma tal closure under drought conditions. It also enriches benzoic acid derivatives and phenols to scavenge ROS and accumulates carbohydrates to maintain cell structural stability (Gowsiga et al., 2025).

### 2.3 Osmotic adjustment and cellular homeostasis

Under drought stress, osmotic adjustment represents a crucial defense mechanism enabling foxtail millet to maintain cell turgor, stabilize macromolecules, and prevent oxidative damage (Gupta et al., 2020). Rather than a single pathway, osmotic balance in foxtail millet results from the coordinated accumulation of compatible solutes, antioxidant enzymes, and stress-protective proteins.

At the biochemical level, foxtail millet tends to accumulate compatible osmolytes such as proline, soluble sugars, γ-aminobutyric acid (GABA), and late embryogenesis abundant (LEA) proteins, which may together contribute to osmotic adjustment by lowering cellular osmotic potential and stabilizing proteins and membranes (Hand et al., 2011; Dien et al., 2019; Gupta et al., 2020; Islam et al., 2024). In foxtail millet, two key proline biosynthetic genes, *SiP5CS1*/*2*, are strongly upregulated under drought, with their activation tightly associated with improved water status (Ajithkumar and Panneerselvam, 2013; Qin et al., 2020; Wang et al., 2023). Soluble sugars derived from starch and sucrose catabolism also play osmo-protective roles under drought (Wang et al., 2023). In addition, SiLEA14 can stabilize cellular homeostasis by buildup osmolytes and conferring stress resistance (Wang et al., 2014). GABA is another important osmo-protectant and signaling molecule in foxtail millet. Recent evidence indicates that exogenous or stress-induced GABA enhances antioxidant enzyme activities, modulates ROS balance, and promotes osmolyte accumulation under drought, thereby improving drought resilience (Islam et al., 2024). In foxtail millet, GABA enhances the activity of antioxidant enzymes, promotes the accumulation of osmotic regulatory substances, reduces the production of reactive oxygen species and membrane lipid peroxidation, and enhances the drought resistance (Yin et al., 2023). Yet, the specific regulatory network of GABA signaling (its receptors, downstream transcriptional targets) remains insufficiently characterized in foxtail millet, representing an important direction for future mechanistic exploration.

### 2.4 Photosynthetic adjustment and energy metabolism

Drought stress alters photosynthesis in foxtail millet to balance energy production with water conservation. Rather than maintaining maximal photosynthetic output, foxtail millet dynamically down-regulates photosynthetic activity to prevent photo-oxidative damage and ensure energy homeostasis under stress. At the morphological level, traits such as reduced leaf area, bulliform cell proliferation, and leaf rolling help minimize transportational water loss and excessive light absorption, thereby reducing photooxidative risk (Wang et al., 2024). At the physiological level, drought stress disrupts photosystem II (PSII) function. The values of F_v_/F_m_, ΦPSII, and photolytic quenching all decrease, accompanied by enhanced non-photochemical quenching (Tang et al., 2017). These coordinated physiological changes, although reducing photosynthetic rate, help prevent photoinhibition and oxidative stress.

At the molecular level, foxtail millet rapidly reorganizes its photosynthetic apparatus and metabolic fluxes. Transcriptomic and proteomic studies reveal downregulation of PSII reaction-center proteins (PsbA, PsbD), the Cytb6f complex, and Calvin cycle enzymes under drought, alongside upregulation of antioxidant and photoprotective protein, such as SiAPX, SiSOD, SiCAT, and SiHSP7 (Pan et al., 2018; Gao et al., 2023; Wang et al., 2024). These changes reflect a coordinated shift from carbon assimilation toward energy stabilization.

Importantly, Existing evidence suggests that foxtail millet activates alternative electron transport pathways, may mediated by SiAOXs, to maintain redox poise and ATP synthesis when CO_2_ assimilation is constrained (Zhang et al., 2024). This flexible redistribution of reducing power likely contributes to the high photosynthetic resilience observed in the foxtail millet. Recent studies further reveal that mitogen-activated protein kinase (MAPK) signaling connects ROS metabolism with photosynthetic regulation. SiMPK6 positively regulates photosynthetic efficiency by enhancing the activities and expression of key antioxidant enzymes, thereby maintaining chloroplast redox balance (Zhu et al., 2025). These networks ensure sustained ROS-redox homeostasis, thereby protecting the photosystems from irreversible oxidative injury.

Energy partitioning and metabolic reallocation are central to drought tolerance in foxtail millet. Under drought stress, rapid accumulation of soluble sugar facilitates osmotic adjustment and sustains mitochondrial respiration when photosynthetic carbon fixation is restricted (Wang et al., 2023). Proteomic evidence further indicates that enzymes involved in malate metabolism and the C4 cycle are upregulated during dehydration, suggesting enhanced NADPH-dependent malate shuttling and efficient energy redistribution (Gao et al., 2023). In contrast, transcriptomic analysis revealed a general downregulation of energy-intensive pathways, implying a metabolic shift that minimizes photorespiratory energy loss under drought stress (Qin et al., 2020). Moreover, SiMYB56 activates lignin biosynthesis genes and enhances ABA signaling, thereby redirecting carbon flow toward structural reinforcement during stress (Xu et al., 2020). Collectively, foxtail millet achieves drought resilience through a multi-layered integration of photochemical control, redox regulation, and metabolic reallocation, ensuring that limited energy resources are dynamically redirected from growth to protection and recovery. However, detailed causal relationships among these processes remain to be clarified, particularly the genetic determinants underlying rapid PSII recovery and alternative electron flow.

### 2.5 Signal perception, integration, and transcriptional reprogramming

Drought stress in foxtail millet triggers a multilayered network of perception, signaling, and transcriptional reprogramming that coordinates rapid physiological and metabolic adjustments (**Figure 1B**). Rather than functioning through a single linear pathway, these responses emerge from dynamic crosstalk among calcium (Ca^2+^), reactive oxygen species (ROS), and hormonal modules, particularly abscisic acid (ABA), that jointly orchestrate drought adaptation.

Signal perception begins with drought-induced cell dehydration, which reduces turgor pressure and activates plasma-membrane mechanosensitive Ca^2+^ channels. This is thought to lead to a transient cytosolic Ca²^+^ influx, which acts as an early secondary messenger that may trigger both ROS and ABA signaling cascades (Yuan et al., 2014; Chen et al., 2020; Gong et al., 2020; Pei et al., 2022). Ca²^+^-binding sensors such as SiCBL5 and calcium-dependent protein kinases (CDPK) decode and relay Ca²^+^ signals downstream. Functional analyses indicate that *SiCDPK24* enhances drought tolerance in transgenic lines, though most evidence currently derives from heterologous expression systems, underscoring the need for *in situ* functional validation in foxtail millet itself (Yu et al., 2018; Yan et al., 2021). In parallel, the m^6^A-reading protein SiYTH1 can stabilize the transcriptional transcripts of *SiCPK6* and *SiOST1*, suggesting that RNA epigenetic modifications may be involved in the precise regulation of the Ca²^+^/ABA signaling pathway (Luo et al., 2023).

ROS act as both stress amplifiers and integrative messengers linking metabolic status to gene regulation. Drought-induced ROS generation in chloroplasts and apoplasts acts dual roles as damaging agents and secondary messengers, they activate redox-sensitive kinases (SiMPK6, SiEULS3) to modulate antioxidant enzyme transcription (e.g., SiAPX, SiCAT, SiSOD) (Pan et al., 2018; Liang et al., 2024; Zhu et al., 2025), trigger MAPK cascades linking stress perception to downstream responses (Mittler, 2002), and serve as a regulatory “bridge” between energy metabolism and hormonal pathways (especially ABA and ethylene), while foxtail millet counteracts ROS accumulation through enhanced activities of enzymatic antioxidants (SOD, POD, CAT, APX) (Pan et al., 2018; Rana et al., 2021; Wang et al., 2023), accumulation of non-enzymatic antioxidants (phenolics, flavonoids, anthocyanins) (Laxa et al., 2019; Wang et al., 2019), reinforcement of the ascorbate-glutathione (AsA-GSH) cycle via SiGR and SiDHAR upregulation, and limitation of mitochondrial ROS generation by alternative oxidases (SiAOXs) (Amoah et al., 2023; Zhang et al., 2024). Additionally, SiNRX1, a redox regulator, has been shown to maintain ROS balance and enhance stress tolerance in Arabidopsis. Consistent with this functional role, the *sinrx1* mutant in foxtail millet exhibits significantly reduced drought resistance (Kneeshaw et al., 2017; Zhang et al., 2022c; Chang et al., 2025). This finding further underscores the critical role of redox regulation in mediating plant adaptation to drought stress.

ABA acts as the central coordinator of hormonal cross-talk under drought, with its biosynthesis genes (*SiNCED1*) and receptors (*PYR/PYLs*) upregulated to reinforce ROS detoxification and stomatal regulation (Waadt et al., 2022; Huang et al., 2023). SiDPY1-SiSAPK6 integrate ABA and osmotic signaling to mediate proline biosynthesis and stomatal control, exemplifying multi-hormone convergence in stress signaling (Zhao et al., 2023). In addition, Brassinosteroids (BRs) antagonize ABA signaling (excessive activation reduces drought tolerance) (Zhao et al., 2023), gibberellin (GA) levels shift with drought severity (Wang et al., 2023), and cytokinin (CK) promote cell division with contrasting regulation between drought-tolerant varieties (DTVs) and drought-sensitive varieties (DSVs) (Tang et al., 2017). Transcriptional regulation forms the downstream core of this signaling crosstalk. Drought-responsive TFs from multiple families act as integrators of hormonal and redox cues, such as NAC, MYB, WRKY, ERF, and bZIP (Bishnoi et al., 2023). SiNAC2, SiNAC18, SiNAC110 respond rapidly to dehydration, linking early perception with transcriptional reprogramming (Lata et al., 2010; Xie et al., 2017). SiMYB56 regulate lignin biosynthesis and secondary wall deposition (Xu et al., 2020). SiWRKY89 enhances stress resilience by activating ABA biosynthetic gene NCED3 and antioxidant systems (Zhang et al., 2022a). ABA Insensitive 5 (ABI5) is a basic leucine zipper transcription factor, and the overexpression of SiABI5.12 confers tolerance to osmotic stress in transgenic Arabidopsis (Wen et al., 2024). Then Abscisic acid-, stress-, and ripening-induced (ASR) transcription factor are actively involved in plant drought tolerance, SiASR1 and SiASR4 enhanced drought tolerances, and decreased ROS production and oxidative damage (Feng et al., 2015; Li et al., 2017). Collectively, these TF families generate an overlapping transcriptional landscape that links hormonal signaling with cellular protection, forming feedback-rich regulatory modules. However, these functional validations were not all conducted in foxtail millet, which became a key experimental obstacle in applying these findings to the verification and breeding processes. In the future, the use of gene editing and allele comparison in different types of foxtail millet varieties will be crucial for confirming the association between these genes and traits.

Beyond transcriptional control, small RNAs and epigenetic modifications add additional regulatory layers. Drought alters miRNA expression in a genotype-dependent manner, with DTVs upregulating miRNAs linked to antioxidant defense and osmotic adjustment (Yadav et al., 2015; Wang et al., 2016). Yet, only the SimiR396d–SiGRF1 module has been experimentally validated in foxtail millet (Zhang et al., 2023). Additionally, drought enriches 24-nt siRNAs involved in DNA methylation and induces the expression of lncRNAs with potential long-range regulatory functions (Qi et al., 2013). However, the functional roles of most miRNAs, siRNAs, and lncRNAs in foxtail millet remain unvalidated. Epigenetic modifications also contribute significantly by regulating gene expression in response to drought stresses (Zhang et al., 2018; Liu et al., 2022b; Sun et al., 2024).In foxtail millet, DNA methylation patterns shift dynamically with drought duration, predominantly at CHG sites, and show genotype-specific plasticity (Wang, 2018). Hydrogen sulfide (H_2_S) is a small signaling molecule. The signaling properties of H_2_S may be attributed to its ability to interact with the thiol groups of protein cysteine residues through post-translational modification such as persulfidation (Thakur and Anand, 2021). In foxtail millet, H_2_S has been suggested to regulate osmotic tolerance, possibly by modulating DNA methylation and DNA methyltransferase (DNMT) activity, thereby reprogramming the expression of TFs. Moreover, foxtail millet exhibits more extensive H_2_S-induced transcriptional changes of DNMTs compared to *Arabidopsis thaliana*, which may contribute to its superior drought resistance (Hao et al., 2020). Histone modifications also play a key role. For example, in the DSV ‘IC41’, SiHDA9 interacts with SiHAT3.1 and SiHDA19 to reduce H3K9 acetylation at drought-responsive promoters, suppressing their expression (Kumar et al., 2023). In contrast, the DTV ‘IC4’ lacks this repression, maintaining higher gene activation under drought stress (Kumar et al., 2023). However, the integration of DNA methylation and histone modification dynamics with phenotypic drought tolerance is still largely unexplored, representing an open frontier in foxtail millet stress biology.

## 3 Genetic and multi-omics resources available to improve drought tolerance in foxtail millet

Foxtail millet exhibits remarkable natural variation and increasingly advanced genomic and omics resources, making it a powerful model for dissecting drought adaptation. Its diverse germplasm, high-quality reference genomes, and integrative omics platforms provide a foundation for linking genetic variation with physiological and agronomic traits under drought. Although significant progress has been made, the systematic utilization of this diversity is still in its infancy. Based on these resources, the recent advancements in multi-omics research are driving a shift from descriptive feature associations to mechanistic and predictive insights into drought adaptability.

### 3.1 Germplasm of foxtail millet and genetic variation for drought tolerance

Foxtail millet has retained a large number of varieties with significant differences in drought tolerance during long-term evolution. Among them, compared with DSVs, DTVs usually maintain higher relative water content, less biomass reduction and more stable root-to-shoot ratio, and have stronger ability to accumulate antioxidants and osmolytes (Yu et al., 2022; Amoah et al., 2023). They also exhibit faster activation of ABA biosynthesis, with hormonal characteristics of higher ABA/GA ratio, more balanced ethylene response and stable cytokinin level (to maintain root growth) (Qin et al., 2020; Wang et al., 2023). Meanwhile, they continuously express drought-responsive TFs and activate epigenetic repressors more strongly, showing a multi-layered adaptive mechanism (Tang et al., 2017; Xiao et al., 2021; Kumar et al., 2023; Chang et al., 2024; Gowsiga et al., 2025). These varieties, which differ in morphology, physiology, and molecular traits, not only reveal the genetic basis of drought tolerance but also serve as core germplasm resources for dissecting foxtail millet’s drought resistance mechanisms and developing improved varieties. Thus, the systematic collection, in-depth exploration, and rational utilization of these resources are crucial for advancing such research and breeding DTVs.

To date, a total of 47,500 foxtail millet germplasms have been collected across different countries, among which China has the largest collection with 26,233 accessions, followed by India (8,506), France (3,500) and Japan (2,531) (Rakkammal et al., 2022). This extensive collection covers rich genetic variation in drought tolerance, laying a solid foundation for screening elite drought-tolerant accessions. Except for natural diversity, mutant libraries such as those generated from the Yugu1 and Ci846 backgrounds using ethyl methanesulfonate (EMS) mutagenesis have expanded the genetic repertoire for forward and reverse genetic studies (Sun et al., 2019; Zhang et al., 2025), enabling fine-scale dissection of drought-related loci.

### 3.2 Genomic and multi-omics resources and advancements in foxtail millet

Genomic and multi-omics resources provide the technical basis for exploring drought resistance mechanisms, with key advancements in genome sequencing, population genetics, and multi-omics integration.

Following the publication of the draft genome in 2012 (Zhang et al., 2012), the complete genome sequence of Yugu1 was obtained in 2023 (He et al., 2023b), greatly advancing molecular research on foxtail millet. Additionally, genomes of its cultivar, landraces, mutants, and wild type (*Setaria viridis*) have been sequenced (Bennetzen et al., 2012; Fang et al., 2016; Yang et al., 2020; He et al., 2023a), providing precise “molecular maps” for drought resistance gene mapping.

In recent years, population genetic research on foxtail millet has advanced rapidly. Key progress includes initial haplotype resolution (Jia et al., 2013), high-depth haplotype mapping of 312 accessions (Li et al., 2021), multi-omics (genomic, transcriptomic, metabolomic) analyses of 398 accessions (Li et al., 2022), and recent large-scale pan-genome studies (He et al., 2023a), extensive investigations into drought-related traits and their underlying genetic mechanisms using germplasm resources. These population genetic studies have enabled efficient mining of drought resistance genes, such as the identification of QTLs (quantitative trait loci) and candidate genes regulating seed water uptake via GWAS (genome-wide association studies, which is a critical step for marker-assisted breeding of DTVs (Li et al., 2023). Furthermore, genomic data can rapidly clarify the evolutionary traits of drought-resistant genes. For instance, haplotypes with enhanced drought tolerance in SiYTH1 and SiSAPK6 are more prevalent in landraces than modern varieties (Luo et al., 2023; Zhao et al., 2023). Meanwhile, these haplotypes can, in principle, be introgressed into elite high-yield backgrounds via marker-assisted selection or edited directly via CRISPR, provided yield penalties are monitored. Thus, population genetic studies provide valuable genetic variation for introgressing drought tolerance into modern varieties.

Additionally, more and more drought-related omics data and integrative omics approaches are revealing cross-scale coordination in foxtail millet’s drought adaptation. Such as, recent transcriptome-wide and weighted gene co-expression network analyses across root, stem and leaf tissues identified more than 13,000 differentially expressed genes under water-limited conditions, uncovering hub regulators linked to cell-cycle, DNA-replication and stress response modules in drought-tolerant versus sensitive genotypes (Zhang et al., 2022b). This finding highlights tissue-specific and genotype-dependent regulatory networks underlying drought tolerance. In parallel, metabolome–transcriptome analyses indicate that accumulation of osmo-protectants such as proline and sucrose correlate strongly with activation of SnRK/AREB signaling module (Qin et al., 2020). This indicates that metabolic adaptation to drought is tightly coupled with key signaling pathways. Moreover, proteomic profiling detected over 320 differentially abundant proteins involved in ROS scavenging, carbon metabolism and photosynthesis under drought in foxtail millet (Pan et al., 2018). These proteins collectively support the maintenance of cellular function under water limitation. Together, these multi-omics datasets support a systems-level model in which drought sensing (via signaling hubs such as Ca²^+^, ABA and ROS) converges with transcriptional, epigenetic and metabolic regulation to determine physiological outcomes. This model not only enhances our understanding of drought adaptation mechanisms but also identifies key targets for genetic manipulation to improve drought tolerance in foxtail millet.

## 4 Discussion and future perspectives

Foxtail millet has two advantages that are stable yield under drought conditions and high efficiency in basic research as a model plant. Although progress has been made in research on the drought resistance mechanism of foxtail millet in recent years, with several genes and regulatory pathways involved in drought adaptation identified, compared with the traditional model plant Arabidopsis, rice, and major crops (wheat and maize) key mechanistic and translational gaps remain that warrant a more prescriptive research focus.

### 4.1 Mechanistic gaps from single-gene studies to new network-level understanding

Most drought-response studies in foxtail millet have so far focused on single genes or individual traits (**Table 1**). A priority for future work is to construct integrative models that capture cross-scale interactions among signals, genes, and physiology. Hormonal cross-talk and root-shoot signaling remain underexplored. While ABA, ROS, and Ca²^+^ pathways are well documented, their dynamic interplay, such as antagonism between ABA and brassinosteroids or synergism with ethylene, remains largely unknown in foxtail millet. For instance, the temporal sequence of Ca²^+^ influx in roots, ROS bursts in leaves, and stomatal closure in the shoot has not yet been resolved with spatio-temporal precision.

**Table 1:** Key genes of foxtail millet involved in drought adaptation and their regulatory roles.

Gene name	Pathway	Physiological and molecular response	Regulation of drought adaptation	References
*SiABI5.12*	ABA	NA	Positive	(Wen et al., 2024)
*SiDPY1*	ABA	Osmotic adjustment	Positive	(Zhao et al., 2023)
*SiSAPK6*	ABA	Osmotic adjustment	Positive	(Zhao et al., 2023)
*SiNCED1*	ABA	Stomatal closure	Positive	(Huang et al., 2023)
*SiWRKY89*	ABA	Osmotic adjustment	Positive	(Zhang et al., 2022a)
		ROS Scavenging		
*SiMYB56*	ABA	Lignin biosynthesis	Positive	(Xu et al., 2020)
*SiASR4*	ABA	Osmotic adjustment	Positive	(Li et al., 2017)
		ROS Scavenging		
*SiARDP*	ABA	ABA-responsive TF	Positive	(Li et al., 2014; Li et al., 2017)
	ROS	Osmotic adjustment		
		ROS scavenging		
*SiMPK6*	ABA	ROS-scavenging	Positive	(Zhu et al., 2025)
	ROS			
*SiYTH1*	ABA	ROS-scavenging	Positive	(Luo et al., 2023)
	ROS	Stomatal closure		
	Ca²^+^			
*SiCBL5*	Ca²	Osmotic balance	Positive	(Yan et al., 2021)
*SiCDPK24*	Ca²	NA	Positive	(Yu et al., 2018)
*SiGRF1*	Ethylene	Root architecture	Negative	(Zhang et al., 2023)
*SimiR396d*	Ethylene	Root architecture	Positive	(Zhang et al., 2023)
*SiEULS3*	ROS	Osmotic adjustment	Positive	(Liang et al., 2024)
		ROS Scavenging		
*SiNRX1*	ROS	Osmotic adjustment ROS Scavenging	Positive	(Zhang et al., 2022c; Chang et al., 2025)
*SiASR1*	ROS	ROS-scavenging	Positive	Feng et al., 2015
*SiEPF2*	NA	Stomatal density	Positive	(Hao et al., 2024; Hao et al., 2025)
*SiLEA14*	NA	Osmotic adjustment	Positive	(Wang et al., 2014)
*SiHDA9*	NA	Epigenetic modifications	Negative	(Kumar et al., 2023)
*SiNAC110*	NA	Osmotic adjustment	Positive	(Xie et al., 2017)

In addition, these advances have also highlighted new mechanisms, such as multi-level signal regulation (Rehaman et al., 2025), hydrogen sulfide (Zhao et al., 2024), and GABA (Islam et al., 2024) as key factors in plant adaptation to drought. However, new mechanisms have not been systematically investigated in foxtail millet. Non-coding RNAs and epigenetic regulation represent another underexplored frontier. Studies in rice and wheat indicate that m^6^A RNA modifications, miRNA modules (e.g., miR396–GRF), and DNA methylation changes may contribute to drought “stress memory”. In foxtail millet, only SimiR396d–SiGRF1 has been functionally verified, primarily through overexpression in foxtail millet seedlings under drought (Zhang et al., 2023), leaving most small RNA–target pairs unvalidated.

Future work should integrate these layers into a multi-scale, heritable drought adaptation network that links root sensing, signal cascades (Ca²^+^/ROS/ABA), and transcriptional–epigenetic control, moving toward predictive modeling of stress resilience. Non-coding RNA and epigenetic layers are another frontier. Studies in other cereals suggest that m^6^A RNA modifications, miRNA modules (e.g., miR396–GRF), and DNA methylation may contribute to stress memory. A “drought memory” phenomenon offers a promising entry point to dissect chromatin–RNA interplay (Amoah et al., 2023). Ultimately, a multi-layer integration linking root sensing, signal transduction (Ca²^+^/ROS/ABA), transcriptional regulation (e.g., SiWRKYs, SibZIPs) (Jia et al., 2024), and physiological outcomes will enable a predictive network model of drought adaptation. However, the organization and heritability of these regulatory layers in foxtail millet under repeated drought cycles remain unexplored.

### 4.2 Technical applications-precision manipulation and field translation

The pan-genome provides a more abundant “gene pool” for the mining of elite alleles. foxtail millet has extensive germplasm resources (Zhao et al., 2025b), which lays a foundation for pan-genome-based allele mining. By integrating technical approaches such as genome-wide association analysis, haplotype mapping, machine learning, quantitative trait locus (QTL) mapping, and meta-QTL (MQTL) to systematically screen for key drought-resistant genes and allelic variants (Loni et al., 2023; Zhu et al., 2024), that underpin crop stress resilience can be recognized.

However, due to limitations in stable genetic transformation technologies for foxtail millet, many drought-resistant genes identified have only been functionally validated in heterologous systems such as Arabidopsis and rice, making it difficult to fully rule out interference from interspecific differences in gene function (Huang et al., 2023; Wen et al., 2024). Notably, breakthroughs have been achieved in foxtail millet transformation technologies in recent years, enabling genetic transformation in several model varieties, though genotype dependence remains (Wang, 2011; Van Eck et al., 2016; Ceasar et al., 2017; Van Eck, 2018; Santos et al., 2020; Sood et al., 2020; Yang et al., 2020; Finley et al., 2021). Future efforts should focus on overcoming this barrier to establish efficient transformation platforms covering more genotype, laying the foundation for dissecting drought-resistant gene functions and their breeding applications.

Future breeding also should, based on the extensive mining of drought-resistant genes in foxtail millet, enhance the application of advanced and precise genetic engineering technologies, such as the CRISPR/Cas9 system and efficient editing strategies to facilitate the creation of beneficial allelic variants (e.g., modifying regulatory alleles of the EPF/ER family that control stomatal density, or miR396–SiGRF1 that regulates root branching) (Zhang et al., 2023; Hao et al., 2025), while avoiding issues like crop growth redundancy and energy waste potentially caused by “constitutive overexpression” in traditional transgenic technologies.

In addition, crop improvement is limited by how plants and their environment interact. However, we don’t have enough research on finding drought-resistant genes and plant lines in foxtail millet. Most studies only test seedlings under one stress, but drought in fields rarely happens alone. So, we should check drought-related genes and plant traits under real, complex field conditions. We also need to balance drought resistance with other important crop traits to turn molecular design ideas into practical foxtail millet breeding.

### 4.3 Extending insights beyond foxtail millet

Foxtail millet offers insights that transcend its own agronomic scope. As a model, it enables more efficient exploration of core regulatory pathways that have evolved independently but gained similar functions across different plant groups, such as SiDPY1–SiSAPK6 module, identified in foxtail millet, responding to osmotic stress in a different manner from the well-characterized RAF-SnRK2 signaling pathway, possibly specific to seed plants (Zhao et al., 2023). Additionally, foxtail millet has developed unique physiological mechanisms, molecular regulatory networks, and genetic adaptation traits during long-term drought adaptation, making it highly valuable for cross-species reference—though this also presents a major challenge for future research. Furthermore, studies on foxtail millet have provided new insights for drought resistance research in other species. Such as drought responses in foxtail millet crosstalking with the circadian (Yi et al., 2022), and H_2_S signals enhancing osmotic stress tolerance via mediating DNA methylation (Hao et al., 2020). Both represent interesting and novel research directions.

Overall, as a C4 crop, foxtail millet further provides a “resilience blueprint” for other C4 species like maize and sorghum; integrating photosynthetic acclimation, dynamic stomatal control, and root energetics here could inform climate-smart breeding strategies.

## 5 Conclusion

Drought remains one of the most severe constraints on global crop productivity, and foxtail millet, with strong drought resistance and suitability for basic research, has become an important subject for exploring crop drought resistance mechanisms. This review has drawn together emerging advances in root plasticity, stomatal regulation, osmotic adjustment, and photosynthetic acclimation, as well as their underlying hormonal, redox, and epigenetic regulators. In the future, it is necessary to establish an integrated multi-omics research framework, strengthen the analysis of mechanism networks, precise gene editing, and cross-species translation of results, to give full plan to foxtail millet’s core role in crop drought resistance research.

## Glossary

ABA: Abscisic acid
ABF: Abscisic acid-responsive element-binding factor
AOX: Alternative oxidase
AP2/ERF: APETALA2/ethylene responsive factor
APX: Ascorbate peroxidase
APX1: Ascorbate peroxidase 1
ARDP: ABA responsive DREB-binding protein
AsA-GSH: Ascorbate-glutathione
ASR: Abscisic acid-, stress-, and ripening-induced
BR: Brassinosteroids
CAT: Catalase
CBL: Calcineurin B-like protein
CDPK/CPK: Calcium-dependent protein kinases
CK: Cytokinin
DHAR: Dehydroascorbate reductase
DNMT: DNA methyltransferase
DPY1: Droopy leaf 1
DSV: Drought-sensitive variety
DTV: Drought-tolerant variety
EPF: Epidermal patterning factor
EULS3: Euonymus lectin 3
GA: Gibberellin
GR: Glutathione reductase
GRF1: Growth regulating factor 1
GRXC7: Glutaredoxin C 7
GULLO4: L-gulonolactone oxidase 4
H₂S: Hydrogen sulfide
H3K9: Methylation of Lys9 in histone H3
HAT3.1: Histone acetyltransferase 3.1
HDA9: Histone deacetylase 9
LEA: Late embryogenesis abundant
lncRNAs: long non-coding RNAs
miRNAs: microRNAs
MPK6: Mitogen-activated protein kinase 6
MYB: v-Myb avian myeloblastosis viral oncogene homolog
NA: Not Available
NAC: NAM, ATAF1/2, and CUC2
NCED1: 9-cis-epoxycarotenoid dioxygenase 1
ncRNAs: Non-coding RNAs
OST1: Open Stomata 1
P5CS1: Δ¹-pyrroline-5-carboxylate synthetase 1
P5CS2: Δ¹-pyrroline-5-carboxylate synthetase 2
PIP: Plasma membrane intrinsic proteins
POD: Peroxidase
PP2C: Protein phosphatase 2C
PYR/PYL: Pyrabactin resistance/PYR-like
RAF: Raf-like kinase
ROS: Reactive oxygen species
SAPK6: Stress-activated protein kinase 6
Si: *Setaria italica*
siRNAs: small interfering RNAs
SnRK2: Sucrose non-fermenting-1-related protein kinase 2
SOD: Superoxide dismutase
TF: Transcription factor
WGCNA: Weighted gene co-expression network analysis
WRKY: WRKY domain-containing protein

## References

[B1] Ajithkumar I. P. Panneerselvam R. (2013). Osmolyte accumulation, photosynthetic pigment and growth of Setaria italica (L.) P. Beauv. under drought stress. Asian Pac J. Reprod. 2, 220–224. doi: 10.1016/s2305-0500(13)60151-7

[B2] Amoah J. N. Adu-Gyamfi M. O. Kwarteng A. O. (2023). Effect of drought acclimation on antioxidant system and polyphenolic content of foxtail millet (Setaria italica L.). Physiol. Mol. Biol. Plants. 29, 1577–1589. doi: 10.1007/s12298-023-01366-w, PMID: 38076760 PMC10709255

[B3] Bennetzen J. L. Schmutz J. Wang H. Percifield R. Hawkins J. Pontaroli A. C. . (2012). Reference genome sequence of the model plant Setaria. Nat. Biotechnol. 30, 555–561. doi: 10.1038/nbt.2196, PMID: 22580951

[B4] Bishnoi A. Jangir P. Soni P. (2023). “ Adaptation of millets to arid land: a special perspective of transcription factors,” in Plant transcription factors (London, United Kingdom: Academic Press), 21–60.

[B5] Ceasar S. A. Baker A. Ignacimuthu S. (2017). Functional characterization of the PHT1 family transporters of foxtail millet with development of a novel Agrobacterium-mediated transformation procedure. Sci. Rep. 7, 14064. doi: 10.1038/s41598-017-14447-0, PMID: 29070807 PMC5656669

[B6] Ceasar S. Penna S. Carvalho C. Jain S. (2025). Millets: Crops for Climate Resilience and for Food and Nutritional Security (Singapore: Springer), 189721.

[B7] Chang X. Zhang S. Cao C. Zhou J. Wang X. Zhang D. . (2024). Transcriptome analysis and characteristics of drought resistance related genes in four varieties of foxtail millet [Setaria italica. Heliyon. 10, e38083. doi: 10.1016/j.heliyon.2024.e38083, PMID: 39364255 PMC11447331

[B8] Chang X. Zhang S. Zhou J. Ren J. Wang X. Song T. . (2025). Transcriptomics-proteomics analysis reveals the role of SiNRX1 in regulating drought stress in foxtail millet (Setaria italica L.). BMC Genom. 26, 920. doi: 10.1186/s12864-025-12123-6, PMID: 41094417 PMC12522487

[B9] Chen K. Gao J. Sun S. Zhang Z. Yu B. Li J. . (2020). BONZAI proteins control global osmotic stress responses in plants. Curr. Biol. 30, 4815–4825.e4. doi: 10.1016/j.cub.2020.09.016, PMID: 33035480

[B10] de Oliveira I. P. Schaaf C. de Setta N. (2024). Drought Responses in Poaceae: Exploring the Core Components of the ABA Signaling Pathway in Setaria italica and Setaria viridis. Plants 13, 1451–1451. doi: 10.3390/plants13111451, PMID: 38891260 PMC11174756

[B11] Dien D. C. Mochizuki T. Yamakawa T. (2019). Effect of various drought stresses and subsequent recovery on proline, total soluble sugar and starch metabolisms in Rice (*Oryza sativa*L.) varieties. Plant Prod. Sci. 22, 530–545. doi: 10.1080/1343943x.2019.1647787

[B12] Doust A. Diao X. (2017). Genetics and Genomics of Setaria. (Cham, Switzerland: Springer). doi: 10.1007/978-3-319-45105-3

[B13] Fang X. Dong K. Wang X. Liu T. He J. Ren R. . (2016). A high density genetic map and QTL for agronomic and yield traits in Foxtail millet [Setaria italica (L.) P. Beauv. BMC Genom 17, 336. doi: 10.1186/s12864-016-2628-z, PMID: 27146360 PMC4857278

[B14] FAO (2023). The impact of disasters on agriculture and food security 2023. (Rome, Italy: FAO). doi: 10.4060/cc7900en

[B15] Feng Z. Xu Z. Sun J. Li L. Chen M. Yang G. . (2015). Investigation of the ASR family in foxtail millet and the role of ASR1 in drought/oxidative stress tolerance. Plant Cell Rep. 35, 115–128. doi: 10.1007/s00299-015-1873-y, PMID: 26441057

[B16] Finley T. Chappell H. Veena V. (2021). *Agrobacterium* -mediated transformation of *setaria viridis*, a model system for cereals and bioenergy crops. Curr. Protoc. 1, e127. doi: 10.1002/cpz1.127, PMID: 33999520

[B17] Gao H. Ge W. Bai L. Zhang T. Zhao L. Li J. . (2023). Proteomic analysis of leaves and roots during drought stress and recovery in Setaria italica L. Front. Plant Sci. 14. doi: 10.3389/fpls.2023.1240164, PMID: 37885665 PMC10598781

[B18] Gong Z. Xiong L. Shi H. Yang S. Herrera-Estrella L. R. Xu G. . (2020). Plant abiotic stress response and nutrient use efficiency. Sci. China Life Sci. 63, 635–674. doi: 10.1007/s11427-020-1683-x, PMID: 32246404

[B19] Gowsiga S. Vijayalakshmi D. Deepika J. Arunkumar P. Sivakumar R. (2025). Stomatal traits, hormone and metabolite profiles governing drought and high temperature stress tolerance in foxtail millet. Russ. J. Plant Physiol. 72, 42. doi: 10.1134/s1021443724609595

[B20] Gupta A. Rico-Medina A. Caño-Delgado A. I. (2020). The physiology of plant responses to drought. Science 368, 266–269. doi: 10.1126/science.aaz7614, PMID: 32299946

[B21] Hand S. C. Menze M. A. Toner M. Boswell L. Moore D. (2011). LEA proteins during water stress: not just for plants anymore. Annu. Rev. Physiol. 73, 115–134. doi: 10.1146/annurev-physiol-012110-142203, PMID: 21034219

[B22] Hao X. Jin Z. Wang Z. Qin W. Pei Y. (2020). Hydrogen sulfide mediates DNA methylation to enhance osmotic stress tolerance in Setaria italica L. Plant Soil. 453, 355–370. doi: 10.1007/s11104-020-04590-5

[B23] Hao J. Kang X. Zhang L. Chen J. Wang D. Dong S. . (2025). CRISPR/Cas9-mediated SiEPF2 mutagenesis attenuates drought tolerance and yield in foxtail millet (Setaria italica). Plant Cell Environ. 48, 6043–6046. doi: 10.1111/pce.15597, PMID: 40302201

[B24] Hao J. Kang X. Zhang L. Zhang J. Wu H. Li Z. . (2024). SiEPFs enhance water use efficiency and drought tolerance by regulating stomatal density in foxtail millet (Setaria italica). J. Integr. Agric. 24, 786–789. doi: 10.1016/j.jia.2024.09.008

[B25] He Q. Tang S. Zhi H. Chen J. Zhang J. Liang H. . (2023a). A graph-based genome and pan-genome variation of the model plant Setaria. Nat. Genet. 55, 1232–1242. doi: 10.1038/s41588-023-01423-w, PMID: 37291196 PMC10335933

[B26] He Q. Wang C. Zhang J. Liang H. Lu Z. Xie K. . (2023b). A complete reference genome assembly for foxtail millet and Setaria-db, a comprehensive database for Setaria. Mol. Plant 17, 219–222. doi: 10.1016/j.molp.2023.12.017, PMID: 38155573

[B27] Hsu P. Dubeaux G. Takahashi Y. Schroeder J. I. (2020). Signaling mechanisms in abscisic acid-mediated stomatal closure. Plant J. 105, 307–321. doi: 10.1111/tpj.15067, PMID: 33145840 PMC7902384

[B28] Hu H. Mauro-Herrera M. Doust A. N. (2018). Domestication and improvement in the model C4 grass, setaria. Front. Plant Sci. 9. doi: 10.3389/fpls.2018.00719, PMID: 29896214 PMC5986938

[B29] Huang Y. Jiao Y. Yang S. Mao D. Wang F. Chen L. . (2023). SiNCED1, a 9-cis-epoxycarotenoid dioxygenase gene in Setaria italica, is involved in drought tolerance and seed germination in transgenic Arabidopsis. Front. Plant Sci. 14. doi: 10.3389/fpls.2023.1121809, PMID: 36968367 PMC10034083

[B30] Islam S. N. Kouser S. Hassan P. Asgher M. Shah A. A. Khan N. A. (2024). Gamma-aminobutyric acid interactions with phytohormones and its role in modulating abiotic and biotic stress in plants. Stress. Biol. 4, 36. doi: 10.1007/s44154-024-00180-y, PMID: 39158750 PMC11333426

[B31] Jangra R. Brunetti S. C. Wang X. Kaushik P. Gulick P. J. Foroud N. A. . (2021). Duplicated antagonistic EPF peptides optimize grass stomatal initiation. Development. 148, dev199780. doi: 10.1242/dev.199780, PMID: 34328169

[B32] Jia X. Gao H. Zhang L. Tang W. Wei G. Sun J. . (2024). Expression of foxtail millet bZIP transcription factor sibZIP67 enhances drought tolerance in arabidopsis. Biomolecules 14, 958. doi: 10.3390/biom14080958, PMID: 39199345 PMC11352937

[B33] Jia G. Huang X. Zhi H. Zhao Y. Zhao Q. Li W. . (2013). A haplotype map of genomic variations and genome-wide association studies of agronomic traits in foxtail millet (Setaria italica). Nat. Genet. 45, 957–961. doi: 10.1038/ng.2673, PMID: 23793027

[B34] Kneeshaw S. Keyani R. Delorme-Hinoux V. Imrie L. Loake G. J. Le Bihan T. . (2017). Nucleoredoxin guards against oxidative stress by protecting antioxidant enzymes. Proc. Natl. Acad. Sci. U.S.A. 114, 8414–8419. doi: 10.1073/pnas.1703344114, PMID: 28724723 PMC5547615

[B35] Kumar V. Singh B. Sharma N. Muthamilarasan M. Sawant S. V. Prasad M. (2023). SiHDA9 interacts with SiHAT3.1 and SiHDA19 to repress dehydration responses through H3K9 deacetylation in foxtail millet. bioRxiv 75, 1098–1111. doi: 10.1101/2023.02.16.528817, PMID: 37889853

[B36] Lata C. Sahu P. P. Prasad M. (2010). Comparative transcriptome analysis of differentially expressed genes in foxtail millet (Setaria italica L.) during dehydration stress. Biochem. Biophys. Res. Commun. 393, 720–727. doi: 10.1016/j.bbrc.2010.02.068, PMID: 20171162

[B37] Laxa M. Liebthal M. Telman W. Chibani K. Dietz K.-J. (2019). The role of the plant antioxidant system in drought tolerance. Antioxidants 8, 94. doi: 10.3390/antiox8040094, PMID: 30965652 PMC6523806

[B38] Li J. Dong Y. Li C. Pan Y. Yu J. (2017). SiASR4, the target gene of siARDP from setaria italica, improves abiotic stress adaption in plants. Front. Plant Sci. 7. doi: 10.3389/fpls.2016.02053, PMID: 28127300 PMC5227095

[B39] Li X. Gao J. Song J. Guo K. Hou S. Wang X. . (2022). Multi-omics analyses of 398 foxtail millet accessions reveal genomic regions associated with domestication, metabolite traits and anti-inflammatory effects. Mol. Plant 15, 367–1383. doi: 10.1016/j.molp.2022.07.003, PMID: 35808829

[B40] Li X. Li Y. Xi R. Hu M. Han Y. Gao J. . (2023). GWAS identifies candidate genes affecting water absorption in foxtail millet seeds. Plant Growth Regul. 102, 545–553. doi: 10.1007/s10725-023-01081-2

[B41] Li C. Wang G. Li H. Wang G. Ma J. Zhao X. . (2021). High-depth resequencing of 312 accessions reveals the local adaptation of foxtail millet. Theor. Appl. Genet. 134, 1303–1317. doi: 10.1007/s00122-020-03760-4, PMID: 33566123

[B42] Li C. Yue J. Wu X. Xu C. Yu J. (2014). An ABA-responsive DRE-binding protein gene from Setaria italica, SiARDP, the target gene of SiAREB, plays a critical role under drought stress. J. Exp. Bot. 65, 5415–5427. doi: 10.1093/jxb/eru302, PMID: 25071221 PMC4157718

[B43] Liang G. Han R. Fan J. Chen Y. Chen Y. Gao C. . (2024). Identification of SiEUL gene family in foxtail millet (Setaria italica L.) and the drought tolerance function of SiEULS3. Plant Growth Regul. 104, 1629–1641. doi: 10.1007/s10725-024-01246-7

[B44] Liu H. Song S. Zhang H. Li Y. Niu L. Zhang J. . (2022a). Signaling transduction of ABA, ROS, and ca2+ in plant stomatal closure in response to drought. IJMS 23, 14824. doi: 10.3390/ijms232314824, PMID: 36499153 PMC9736234

[B45] Liu Y. Wang J. Liu B. Xu Z. (2022b). Dynamic regulation of DNA methylation and histone modifications in response to abiotic stresses in plants. JIPB 64, 2252–2274. doi: 10.1111/jipb.13368, PMID: 36149776

[B46] Liu T. Ye N. Song T. Cao Y. Gao B. Zhang D. . (2018). Rhizosheath formation and involvement in foxtail millet (*Setaria italica*) root growth under drought stress. JIPB 61, 449–462. doi: 10.1111/jipb.12716, PMID: 30183129

[B47] Loni F. Ismaili A. Nakhoda B. Darzi Ramandi H. Shobbar Z.-S. (2023). The genomic regions and candidate genes associated with drought tolerance and yield-related traits in foxtail millet: an integrative meta-analysis approach. Plant Growth Regul. 101, 169–185. doi: 10.1007/s10725-023-01010-3

[B48] Luo W. Tang Y. Li S. Zhang L. Liu Y. Zhang R. . (2023). The m6A reader SiYTH1 enhances drought tolerance by affecting the mRNA stability of genes related to stomatal closure and ROS scavenging in Setaria italica. JIPB 65, 2569–2586. doi: 10.1111/jipb.13575, PMID: 37861067

[B49] Ma X. Qin J. Guo B. Jia M. Li G. Li L. . (2025). Genome-wide identification of the PYL gene family and functional validation of SiPYL3 in foxtail millet (Setaria italica). Plant Physiol. Biochem. 227, 110118–110118. doi: 10.1016/j.plaphy.2025.110118, PMID: 40483762

[B50] Mekonnen M. M. Hoekstra A. Y. (2016). Four billion people facing severe water scarcity. Sci. Adv. 2, e1500323. doi: 10.1126/sciadv.1500323, PMID: 26933676 PMC4758739

[B51] Mittler R. (2002). Oxidative stress, antioxidants and stress tolerance. Trends. Plant Sci. 7, 405–410. doi: 10.1016/s1360-1385(02)02312-9, PMID: 12234732

[B52] Nadeem F. Ahmad Z. Ul Hassan M. Wang R. Diao X. Li X. (2020). Adaptation of foxtail millet (Setaria italica L.) to abiotic stresses: A special perspective of responses to nitrogen and phosphate limitations. Front. Plant Sci. 11. doi: 10.3389/fpls.2020.00187, PMID: 32184798 PMC7058660

[B53] Nations U. (2022). Drought in Numbers. Bonn: UNCCD Publication.

[B54] Niu H. Guo W. (2022). Effects of herbicide mixture and dosages on agronomic characters and yield of millet k864 - 3. Shaanxi J. Agric. Sci. 68, 12–15.

[B55] Pan J. Li Z. Wang Q. Garrell A. K. Liu M. Guan Y. . (2018). Comparative proteomic investigation of drought responses in foxtail millet. BMC Plant Biol. 18, 315. doi: 10.1186/s12870-018-1533-9, PMID: 30497407 PMC6267058

[B56] Pardo J. VanBuren R. (2021). Evolutionary innovations driving abiotic stress tolerance in C4 grasses and cereals. Plant Cell 33, 3391–3401. doi: 10.1093/plcell/koab205, PMID: 34387354 PMC8566246

[B57] Pei S. Liu Y. Li W. Krichilsky B. Dai S. Wang Y. . (2022). OSCA1 is an osmotic specific sensor: a method to distinguish Ca^2+^-mediated osmotic and ionic perception. New Phytol. 235, 1665–1678. doi: 10.1111/nph.18217, PMID: 35527515

[B58] Qi X. Xie S. Liu Y. Yi F. Yu J. (2013). Genome-wide annotation of genes and noncoding RNAs of foxtail millet in response to simulated drought stress by deep sequencing. Plant Mol. Biol. 83, 459–473. doi: 10.1007/s11103-013-0104-6, PMID: 23860794

[B59] Qie L. Jia G. Zhang W. Schnable J. Shang Z. Li W. . (2014). Mapping of Quantitative Trait Locus (QTLs) that Contribute to Germination and Early Seedling Drought Tolerance in the Interspecific Cross Setaria italica×Setaria viridis. PLoS One 9, e101868. doi: 10.1371/journal.pone.0101868, PMID: 25033201 PMC4102488

[B60] Qin L. Chen E. Li F. Yu X. Liu Z. Yang Y. . (2020). Genome-Wide Gene Expression Profiles Analysis Reveal Novel Insights into Drought Stress in Foxtail Millet (Setaria italica L.). IJMS 21, 8520–8520. doi: 10.3390/ijms21228520, PMID: 33198267 PMC7696101

[B61] Qin H. Xiao M. Li Y. Huang R. (2024). Ethylene modulates rice root plasticity under abiotic stresses. Plants 13, 432–432. doi: 10.3390/plants13030432, PMID: 38337965 PMC10857340

[B62] Rakkammal K. Priya A. Pandian S. Maharajan T. Rathinapriya P. Satish L. . (2022). Conventional and omics approaches for understanding the abiotic stress response in cereal crops—An updated overview. Plants 11, 2852. doi: 10.3390/plants11212852, PMID: 36365305 PMC9655223

[B63] Rana S. Pramitha L. Muthamilarasan M. (2021). “ Genomic designing for abiotic stress tolerance in foxtail millet (Setaria italica L.),” in Genomic Designing for Abiotic Stress Resistant Cereal Crops ( Springer International Publishing, Cham), 255–289. doi: 10.1007/978-3-030-75875-2_7

[B64] Rehaman A. Khan S. Rawat B. Gaira K. S. Asgher M. Semwal P. . (2025). Mechanistic Insights into Plant Drought Tolerance: A Multi-level Perspective. Deleted J. 77, 53. doi: 10.1007/s10343-025-01115-x

[B65] Santos C. M. Romeiro D. Silva J. P. Basso M. F. Molinari H. B. C. Centeno D. C. (2020). An improved protocol for efficient transformation and regeneration of Setaria italica. Plant Cell Rep. 39, 501–510. doi: 10.1007/s00299-019-02505-y, PMID: 31915913

[B66] Schroeder J. I. Kwak J. M. Allen G. J. (2001). Guard cell abscisic acid signalling and engineering drought hardiness in plants. Nature 410, 327–330. doi: 10.1038/35066500, PMID: 11268200

[B67] Sood P. Singh R. K. Prasad M. (2020). An efficient Agrobacterium-mediated genetic transformation method for foxtail millet (Setaria italica L.). Plant Cell Rep. 39, 511–525. doi: 10.1007/s00299-019-02507-w, PMID: 31938834

[B68] Sun C. Guo Z. Lu C. (2011). Study on Photosynthetic Characteristics and Water Use Efficiency in 4 Crops. J. Shanxi Agric. Sci. 39, 791–793. doi: 10.3969/j.issn.1002-2481.2011.08.05

[B69] Sun J. Luu N. S. Chen Z. Chen B. Cui X. Wu J. . (2019). Generation and Characterization of a Foxtail Millet (Setaria italica) Mutant Library. Front. Plant Sci. 10. doi: 10.3389/fpls.2019.00369, PMID: 31001298 PMC6455083

[B70] Sun Y. Wang X. Di Y. Li J. Li K. Wei H. . (2024). Systematic Analysis of DNA Demethylase Gene Families in Foxtail Millet (Setaria italica L.) and Their Expression Variations after Abiotic Stresses. Int. J. Mol. Sci. 25, 4464–4464. doi: 10.3390/ijms25084464, PMID: 38674049 PMC11050331

[B71] Tang S. Li L. Wang Y. Chen Q. Zhang W. Jia G. . (2017). Genotype-specific physiological and transcriptomic responses to drought stress in Setaria italica (an emerging model for Panicoideae grasses). Sci. Rep. 7, 10009. doi: 10.1038/s41598-017-08854-6, PMID: 28855520 PMC5577110

[B72] Tang R.-J. Wang C. Li K. Luan S. (2020). The CBL–CIPK Calcium Signaling Network: Unified Paradigm from 20 Years of Discoveries. Trends. Plant Sci. 25, 604–617. doi: 10.1016/j.tplants.2020.01.009, PMID: 32407699

[B73] Thakur M. Anand A. (2021). Hydrogen sulfide: An emerging signaling molecule regulating drought stress response in plants. Physiol. Plant 172, 1227–1243. doi: 10.1111/ppl.13432, PMID: 33860955

[B74] Upadhyaya H. D. Vetriventhan M. Deshpande S. P. Sivasubramani S. Wallace J. G. Buckler E. S. . (2015). Population Genetics and Structure of a Global Foxtail Millet Germplasm Collection. Plant Genome 8, plantgenome2015.07.0054. doi: 10.3835/plantgenome2015.07.0054, PMID: 33228275

[B75] Van Eck J. (2018). The Status of Setaria viridis Transformation: Agrobacterium-Mediated to Floral Dip. Front. Plant Sci. 9. doi: 10.3389/fpls.2018.00652, PMID: 29887870 PMC5981604

[B76] Van Eck J. Swartwood K. Pidgeon K. Maxson-Stein K. (2016). “ Agrobacterium tumefaciens-Mediated Transformation of Setaria viridis,” in Genetics and Genomics of Setaria ( Springer International Publishing, Cham), 343–356. doi: 10.1007/978-3-319-45105-3_20

[B77] Veldkamp T. I. E. Wada Y. Aerts J. C. J. H. Ward P. J. (2016). Towards a global water scarcity risk assessment framework: incorporation of probability distributions and hydro-climatic variability. Environ. Res. Lett. 11, 024006–024006. doi: 10.1088/1748-9326/11/2/024006

[B78] Waadt R. Seller C. A. Hsu P.-K. Takahashi Y. Munemasa S. Schroeder J. I. (2022). Plant hormone regulation of abiotic stress responses. Nat. Rev. | Mol. Cell Biol. 23, 680–694. doi: 10.1038/s41580-022-00479-6, PMID: 35513717 PMC9592120

[B79] Wang M.-Z. (2011). Culturing of immature inflorescences and Agrobacterium-mediated transformation of foxtail millet (Setaria italica). Afr. J. Biotechnol. 10, 16466–16479. doi: 10.5897/ajb10.2330

[B80] Wang J. (2018). Study on the Effect of DNA Cytosine Methylation Variations induced by Drought Stess in Millet (Liaoning, China: Liaoning University).

[B81] Wang Z. Ding Y. He J. Yu J. (2004). An updating analysis of the climate change in China in recent 50 years. Acta Meteorologica Sin. 62, 228–236.

[B82] Wang M. Li P. Li C. Pan Y. Jiang X. Zhu D. . (2014). SiLEA14, a novel atypical LEA protein, confers abiotic stress resistance in foxtail millet. BMC Plant Biol. 14, 290. doi: 10.1186/s12870-014-0290-7, PMID: 25404037 PMC4243736

[B83] Wang Y. Li L. Tang S. Liu J. Zhang H. Zhi H. . (2016). Combined small RNA and degradome sequencing to identify miRNAs and their targets in response to drought in foxtail millet. BMC Genet. 17, 57. doi: 10.1186/s12863-016-0364-7, PMID: 27068810 PMC4828802

[B84] Wang X. Liu H. Yu F. Hu B. Jia Y. Sha H. . (2019). Differential activity of the antioxidant defence system and alterations in the accumulation of osmolyte and reactive oxygen species under drought stress and recovery in rice (Oryza sativa L.) tillering. Sci. Rep. 9, 1038. doi: 10.1038/s41598-019-44958-x, PMID: 31189967 PMC6561971

[B85] Wang J. Sun Z. Tian L. Sun W. Wang X. Wang Z. . (2024). Transcriptome-metabolome and anatomy conjoint analysis of vital component change of photosynthesis of Foxtail millet under different drought conditions. J. Integr. Agric. 24, 4588–4612. doi: 10.1016/j.jia.2024.04.001

[B86] Wang J. Sun Z. Wang X. Tang Y. Li X. Ren C. . (2023). Transcriptome-based analysis of key pathways relating to yield formation stage of foxtail millet under different drought stress conditions. Front. Plant Sci. 13. doi: 10.3389/fpls.2022.1110910, PMID: 36816479 PMC9937063

[B87] Wen Y. Zhao Z. Cheng L. Zhou S. An M. Zhao J. . (2024). Genome-wide identification and expression profiling of the ABI5 gene family in foxtail millet (Setaria italica). BMC Plant Biol. 24, 164. doi: 10.1186/s12870-024-04865-4, PMID: 38431546 PMC10908088

[B88] Wilkins K. A. Matthus E. Swarbreck S. M. Davies J. M. (2016). Calcium-Mediated Abiotic Stress Signaling in Roots. Front. Plant Sci. 7. doi: 10.3389/fpls.2016.01296, PMID: 27621742 PMC5002411

[B89] Xiao J. Sun Z. Chen G. Liu Z. Xin Z. Kong F. (2021). Evaluation of drought tolerance in different genotypes of foxtail millet during the entire growth period. Agron. J. 114, 340–355. doi: 10.1002/agj2.20908

[B90] Xie L. Chen M. Min D. Feng L. Xu Z. Zhou Y. . (2017). The NAC-like transcription factor SiNAC110 in foxtail millet (Setaria italica L.) confers tolerance to drought and high salt stress through an ABA independent signaling pathway. J. Integr. Agr. 16, 559–571. doi: 10.1016/s2095-3119(16)61429-6

[B91] Xiong Y. Song X. Mehra P. Yu S. Li Q. Tashenmaimaiti D. . (2025). ABA-auxin cascade regulates crop root angle in response to drought. Curr. Biol. 35, 542–553.e4. doi: 10.1016/j.cub.2024.12.003, PMID: 39798563

[B92] Xu W. Tang W. Wang C. Ge L. Sun J. Qi X. . (2020). SiMYB56 confers drought stress tolerance in transgenic rice by regulating lignin biosynthesis and ABA signaling pathway. Front. Plant Sci. 11. doi: 10.3389/fpls.2020.00785, PMID: 32625221 PMC7314972

[B93] Yadav A. Khan Y. Prasad M. (2015). Dehydration-responsive miRNAs in foxtail millet: genome-wide identification, characterization and expression profiling. Planta 243, 749–766. doi: 10.1007/s00425-015-2437-7, PMID: 26676987

[B94] Yan J. Yang L. Liu Y. Zhao Y. Han T. Miao X. . (2021). Calcineurin B-like protein 5 (SiCBL5) in Setaria italica enhances salt tolerance by regulating Na+ homeostasis. Crop J. 10, 234–242. doi: 10.1016/j.cj.2021.06.006

[B95] Yang X. Tian Q. Yan J. Chen Y. (2022). Characterizing root morphological traits in 65 genotypes of foxtail millet (Setaria italica L.) from four different ecological regions in China. Agronomy. (Basel). 12, 1472–1472. doi: 10.3390/agronomy12061472

[B96] Yang Z. Zhang H. Li X. Shen H. Gao J. Hou S. . (2020). A mini foxtail millet with an Arabidopsis-like life cycle as a C4 model system. Nat. Plants 6, 1167–1178. doi: 10.1038/s41477-020-0747-7, PMID: 32868891

[B97] Yi F. Huo M. Li J. Yu J. (2022). Time-series transcriptomics reveals a drought-responsive temporal network and crosstalk between drought stress and the circadian clock in foxtail millet. Plant J. 110, 1213–1228. doi: 10.1111/tpj.15725, PMID: 35262997

[B98] Yin M. Wang Y. Wen Y. Su J. Fan D. Zhao J. . (2023). Physiological mechanism of γ-aminobutyric acid priming improves foxtail millet seed germination under drought stress. Zhiwu Shengli Xuebao 59, 923–931. doi: 10.13592/j.cnki.ppj.100556

[B99] Yu G. Liu P. Hao H. Cui H. Guo A. Li M. (2022). Regulation mechanism of drought resistance in different genotypes of foxtail millet. J. Plant Nutr. Fertil. 28, 157–167. doi: 10.11674/zwyf.2021134

[B100] Yu T.-F. Zhao W.-Y. Fu J.-D. Liu Y.-W. Chen M. Zhou Y.-B. . (2018). Genome-wide analysis of CDPK family in foxtail millet and determination of siCDPK24 functions in drought stress. Front. Plant Sci. 9. doi: 10.3389/fpls.2018.00651, PMID: 30093908 PMC6071576

[B101] Yuan F. Yang H. Xue Y. Kong D. Ye R. Li C. . (2014). OSCA1 mediates osmotic-stress-evoked Ca2+ increases vital for osmosensing in Arabidopsis. Nature 514, 367–371. doi: 10.1038/nature13593, PMID: 25162526

[B102] Zhai P. Liu J. (2012). Extreme weather/climate events and disaster prevention and mitigation under global warming background. Strategic Study CAE 14, 55–63 + 84.

[B103] Zhang H. Lang Z. Zhu J.-K. (2018). Dynamics and function of DNA methylation in plants. Nat. Rev. Mol. Cell Biol. 19, 489–506. doi: 10.1038/s41580-018-0016-z, PMID: 29784956

[B104] Zhang H. Liang H. Zhi H. Yuan D. Yao Q. Zhang R. . (2025). An efficient target-mutant screening platform of model variety Ci846 facilitates genetic studies of Setaria. Plant Biotechnol. J. 23, 1413–1415. doi: 10.1111/pbi.14594, PMID: 40029790 PMC12018841

[B105] Zhang J. Liu T. Fu J. Zhu Y. Jia J. Zheng J. . (2007). Construction and application of EST library from Setaria italica in response to dehydration stress. Genomics 90, 121–131. doi: 10.1016/j.ygeno.2007.03.016, PMID: 17498921

[B106] Zhang G. Liu X. Quan Z. Cheng S. Xu X. Pan S. . (2012). Genome sequence of foxtail millet (Setaria italica) provides insights into grass evolution and biofuel potential. Nat. Biotechnol. 30, 549–554. doi: 10.1038/nbt.2195, PMID: 22580950

[B107] Zhang H. Luo Y. Wang Y. Zhao J. Wang Y. Li Y. . (2024). Genome-Wide Identification and Characterization of Alternative Oxidase (AOX) Genes in Foxtail Millet (Setaria italica): Insights into Their Abiotic Stress Response. Plants 13, 2565–2565. doi: 10.3390/plants13182565, PMID: 39339540 PMC11434880

[B108] Zhang L. Ren Y. Wan Y. Xu Q. Yang G. Zhang S. . (2021). SiCEP3, a C-terminally encoded peptides from*Setaria italica*, promotes ABA import and signaling pathway. J. Exp. Bot. 72, 6260–6273. doi: 10.1101/2021.01.30.428944 34097059

[B109] Zhang Y. Xiao T. Yi F. Yu J. (2023). SimiR396d targets SiGRF1 to regulate drought tolerance and root growth in foxtail millet. Plant Sci. 326, 111492. doi: 10.1016/j.plantsci.2022.111492, PMID: 36243168

[B110] Zhang S. Yu Y. Song T. Zhang M. Li N. Yu M. . (2022c). Genome-wide identification of foxtail millet’s TRX family and a functional analysis of SiNRX1 in response to drought and salt stresses in transgenic Arabidopsis. Front. Plant Sci. 13. doi: 10.3389/fpls.2022.946037, PMID: 36226299 PMC9549295

[B111] Zhang A. Zhang L. Guo E. Wang R. Li Q. Guo S. . (2022a). Setaria italica SiWRKY89 enhances drought tolerance in Arabidopsis. Plant Growth Regul. 99, 125–135. Available online at: https://xueshu.baidu.com/usercenter/paper/show?paperid=163a04j0qb4900a0tu6q0gf04f388871 (Accessed October 15, 2022).

[B112] Zhang R. Zhi H. Li Y. Guo E. Feng G. Tang S. . (2022b). Response of multiple tissues to drought revealed by a weighted gene co-expression network analysis in foxtail millet [Setaria italica (L.) P. Beauv. Front. Plant Sci. 12. doi: 10.3389/fpls.2021.746166, PMID: 35095942 PMC8790073

[B113] Zhao Y. Liu J. Kuerban Z. Wang H. Yang B. Wang H.-J. . (2025b). Drought tolerance evaluation and classification of foxtail millet core germplasms using comprehensive tolerance indices. Life 15, 1485. doi: 10.3390/life15091485, PMID: 41010427 PMC12471440

[B114] Zhao X. Liu H. Liu E. Huang M. (2025a). Analysis of the difference in water consumption and water use efficiency among different drought-resistant foxtail millet varieties. J. Water. Clim. Change 16, 2069–2083. doi: 10.2166/wcc.2025.780

[B115] Zhao M. Zhang Q. Liu H. Tang S. Shang C. Zhang W. . (2023). The osmotic stress–activated receptor-like kinase DPY1 mediates SnRK2 kinase activation and drought tolerance in Setaria. Plant Cell 35, 3782–3808. doi: 10.1093/plcell/koad200, PMID: 37462269 PMC10533336

[B116] Zhao J. Zhang S. Yang X. Feng K. Wang G. Shi Q. . (2024). Hydrogen sulfide increases drought tolerance by modulating carbon and nitrogen metabolism in foxtail millet seedlings. Agronomy. (Basel). 14, 1080–1080. doi: 10.3390/agronomy14051080

[B117] Zhu D. Hu X. Wang H. Zhang Y. Li X. Song W. . (2025). Insight into the Functional Role of SiMPK6 in Stress Response and Photosynthetic Efficiency in Setaria italica. Plants 14, 1960–1960. doi: 10.3390/plants14131960, PMID: 40647969 PMC12251781

[B118] Zhu C. Zhao L. Zhao S. Niu X. Li L. Gao H. . (2024). Utilizing machine learning and bioinformatics analysis to identify drought-responsive genes affecting yield in foxtail millet. Int. J. Biol. Macromol 277, 134288. doi: 10.1016/j.ijbiomac.2024.134288, PMID: 39079238

